# Supplementary material for: Peste des Petits Ruminants (PPR) Vaccination Cost Estimates in Burkina Faso
Source: Animals (Basel). 2022 Aug 22;12(16):2152. doi: 10.3390/ani12162152 (PMC9405497; doi:10.3390/ani12162152)
Supplement: Supplementary file 1 [file animals-12-02152-s001.zip › Figure S2.pdf]

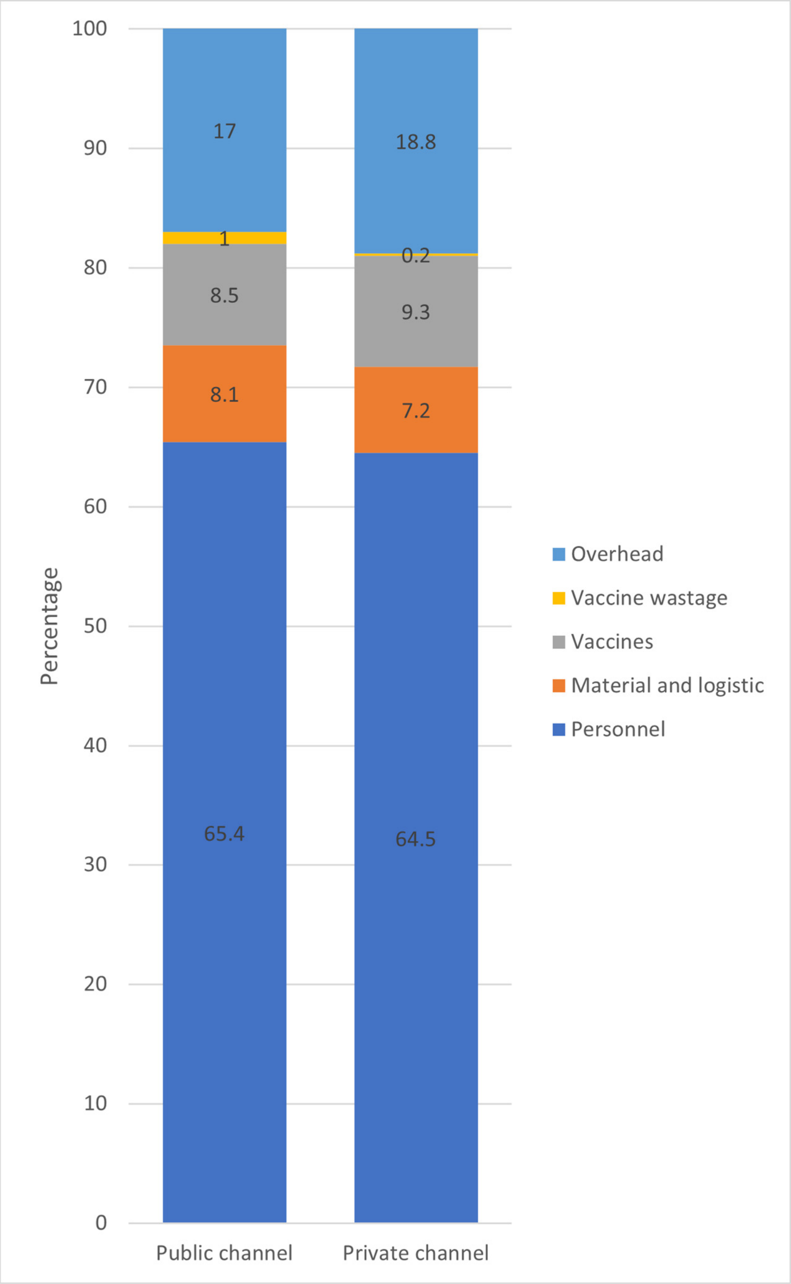

Figure S2: Percentage contribution of various inputs to PPR vaccination cost in public and private distribution channels in Burkina Faso in 2020
