# Supplementary material for: Peste des Petits Ruminants (PPR) Vaccination Cost Estimates in Burkina Faso
Source: Animals (Basel). 2022 Aug 22;12(16):2152. doi: 10.3390/ani12162152 (PMC9405497; doi:10.3390/ani12162152)
Supplement: Supplementary file 1 [file animals-12-02152-s001.zip › Tables S10-S12.pdf]

Cost calculation details by inputs

Note: unit cost= total cost/number of animals vaccinated

- *Personnel cost*
- *Vaccines*
- *Material and logistic*
- *Overhead*
- *Vaccine wastage*

Table S10. Personnel cost calculation details

Vaccine distribution level		Central	Regional	Provincial	Communal	Private vets
Personnel cost = sub total1 + sub total2 + sub total3 + sub total4 + sub total5 + sub total6 + sub total7		48,169,833	457,667	383,750	1,068,833	5,225,767
For vaccine transportation	*Sub total1 = (c + d) *a*b*e*	*764,000*	*57,667*	*33,333*	*9,500*	*56,667*
	No of people (a)	2	2	2	1	1
	No of days of transportation (b)	12	1	1	0.5	1
	Per diem (c)	23,500	20,500	0	0	15,000
	Daily salary (d)	8,333	8,333	8,333	6,333	13,333
	Supply number (e)	1	1	2	3	2
For vaccine storage	*Sub total2*	*0*	*0*	*0*	*0*	*0*
For vaccine field delivery	*Sub total3 (communal) = (i*h*g*f) + (j*g*f)*	0	0	0	870,000	n/a
	*Sub total3 (private vets) = (f*g*h*i) + j*				n/a	4,213,600
	No of vaccination teams (f)	n/a	n/a	n/a	1	6
	No of people per team (g)	n/a	n/a	n/a	2	2
	No of working days per team (h)	n/a	n/a	n/a	45	48
	Daily salary (i)	n/a	n/a	n/a	6,333	0
	Per diem (j)	n/a	n/a	n/a	150,000	4,213,600
For sensitization	*Sub total4 (central)= n + o*k*m*l + p*m*	*14,085,500*	n/a	n/a	n/a	n/a
	Sub total4 (provincial)= k*l*m*o + n*m*k + p*m	n/a	n/a	*113,333*	0	0

	Sub total4 (communal, private vets)= k*l*m*o + n*k + p*m	*0*	*0*	*0*	*189,333*	*372,167*
	No of people involved (k)	100	n/a	2	2	2
	No of days per session (l)	1	n/a	1	1	1
	No of sessions (m)	1	n/a	2	8	13
	Per diem (n)	9,775,500	n/a	20,000	30,000	55,000
	Daily salary (o)	10,000	8,333	8,333	6,333	6,333
	Refreshment per session (p)	3,310,000	0	0	3,500	7,500
For supervision	*Sub total5 (central)* = s*q*u*t + r*q*u*t	*4,440,000*				
	Sub total5 (others) = s + q*t*u*r		*275,000*	*143,333*	*0*	*133,333*
	No of days of supervision (q)	5	1	1	n/a	1
	Daily salary (r)	10,000	8,333	8,333	n/a	13,333
	Per diem (s)	27,000	150,000	60,000	n/a	0
	No of people involved (t)	4	3	2	n/a	1
	No of supervision sessions (u)	6	5	5	n/a	10
For training and meetings	*Sub total6 = b1+g1+c1*a1*e1+h1*f1+d1+i1*	*24,450,333*	0	0	0	0
	Review workshop		n/a	n/a	n/a	n/a
	No of people (a1)	80	n/a	n/a	n/a	n/a
	Per diem (b1)	2,877,000	n/a	n/a	n/a	n/a
	Daily salary (c1)	10,000	n/a	n/a	n/a	n/a
	Refreshment (d1)	594,000	n/a	n/a	n/a	n/a
	No of days (e1)	2	n/a	n/a	n/a	n/a
	Animal marking training		n/a	n/a	n/a	n/a
	No of people (f1)	163	n/a	n/a	n/a	n/a
	Perdiem (g1)	16,369,000	n/a	n/a	n/a	n/a
	Daily salary (h1)	6,333	n/a	n/a	n/a	n/a
	Refreshment (i1)	1,978,000	n/a	n/a	n/a	n/a
For coordination	*Sub total7= n1*k1*j1+m1*l1*k1*j1 + (o1 + p1)*q1*n1*	*4,430,000*	*125,000*	*93,750*	*0*	*450,000*
	No of people (j1)	4	2	1	n/a	1
	No of working days (k1)	40	20	30	n/a	45
	Percentage time spent on vaccination (l1)	0.5	0.375	0.375	n/a	0.75

	Daily salary (m1)	10,000	8,333	8,333	n/a	13,333
	Reporting cost		n/a	n/a	n/a	n/a
	No of people (n1)	11	n/a	n/a	n/a	n/a
	Per dem (o1)	5,000	n/a	n/a	n/a	n/a
	Daily salary (p1)	10,000	n/a	n/a	n/a	n/a
	No of working days (q1)	22	n/a	n/a	n/a	n/a

Table S11. Vaccine cost calculation details

Vaccine distribution level	Central	Regional	Provincial	Communal	Private vets
*Total cost = a * b*	*105,846,022*	*0*	*0*	*0*	*0*
Unit dose cost (a)	23.81	n/a	n/a	n/a	n/a
Number of doses used (b)	4,445,444	234,122	60,888	11,191	84,272

Table S12. Material and logistic cost calculation details

Vaccine distribution level		Central	Regional	Provincial	Communal	Private vets
Material and logistic = sub total1 + sub total2 + sub total3 + sub total4 + sub total5 + sub total6 + sub total7		12,559,963	109,722	142,014	101,635	530,464
For vaccine transportation	*Sub total1*= a/(360*b)*c*d + h + e/(360*f)*g*h	*333,333*	*13,889*	*27,778*	*38,751*	*241,673*
	Mean of transportation price (a)	50,000,000	25,000,000	25,000,000	1,500,000	1,500,000
	Mean of transportation ULY (b)	5	5	5	5	5
	No of days of transportation (c)	12	1	1	0.5	1
	Supply number (d)	1	1	2	3	2
	Motorbike price (e)	1	1	1	1,500,000	1,500,000
	Motorbike ULY (f)	1	1	1	5	5
	No of days of transportation in the field (g)	n/a	n/a	n/a	45	48
	No of vaccination teams (h)	n/a	n/a	n/a	1	6
For vaccine storage	*Sub total2 = =j/(360*k)*i*l*m +o/(360*p)*n*s+q/(360*r)*s*	*4,575,000*	*22,222*	*13,889*	*19,109*	*40,023*
	Cold room number (i)	2	n/a	n/a	n/a	n/a
	Cold room building cost (j)	25,000,000	n/a	n/a	n/a	n/a
	Cold room ULY (k)	10	n/a	n/a	n/a	n/a
	Cold room storage time in days (l)	360	n/a	n/a	n/a	n/a
	Cold room percentage of use (m)	0.9	n/a	n/a	n/a	n/a
	Refrigerator number (n)	1	2	1	1	2
	Refrigerator price (o)	1,500,000	500,000	500,000	500,000	500,000
	Refrigerator ULY (p)	5	5	5	5	5
	Gas bottle price (q)	0	0	0	17,500	17,500
	Gas bottle ULY (r)	1	1	1	3	3
	Refrigerator storage time (s)	90	40	50	65	70
For vaccine field delivery	*Sub total3 =* c1/*d1 + e1/f1 + g1/h1 + i1/j1)*m1 + (k1/(360*l1))*n1*a1*	*0*	*0*	*0*	*37108*	*220227*
	No of vaccination teams (a1)	n/a	n/a	n/a	1	6
	No of people per team (b1)	n/a	n/a	n/a	2	2

	Vaccination syringe price (c1)	n/a	n/a	n/a	30,000	30,000
	No of doses supported (d1)	n/a	n/a	n/a	15,000	25,000
	Needle price (e1)	n/a	n/a	n/a	250	250
	No of doses supported (f1)	n/a	n/a	n/a	1,000	1,000
	Spare glass price (g1)	n/a	n/a	n/a	3,000	3,000
	No of doses supported (h1)	n/a	n/a	n/a	10,000	5,000
	Marking clamp (i1)	n/a	n/a	n/a	10,000	10,000
	No of animal supported (j1)	n/a	n/a	n/a	20,000	20,000
	Cold box price (k1)	n/a	n/a	n/a	71,420	20,000
	Cold box ULY (l1)	n/a	n/a	n/a	3	3
	No of animal vaccinated (m1)	4,445,444	234,122	60,888	11,191	84,272
	No of days of work per team (n1)	40			45	48
For sensitization	Sub total4 = p1/(360*q1)*r1*o1 + s1	2,695,556	0	27,778	6,667	10,833
	No of sessions (o1)	1		2	8	13
	Mean of transportation price (p1)	50,000,000	25,000,000	25,000,000	1,500,000	1,500,000
	Mean of transportation ULY (q1)	5		5	5	5
	Mean of transportation number (r1)	38	0	1	1	1
	Other material cost (s1)	1,640,000	0	0	0	0
For supervision	Sub total5 = v1/(360*w1)*u1*t1	833,333	69,444	69,444	0	8,333
	No of days (t1)	5	1	1	n/a	1
	No of sessions (u1)	6	5	5	n/a	10
	Mean of transportation price (v1)	50,000,000	25,000,000	25,000,000	n/a	1,500,000
	Mean of transportation ULY (w1)	5	5	5	n/a	5
For training and meetings	Sub total6 = a2 + b2/(360*f2)*e2*c2 + g2 + h2/(360*k2)*j2*i2	4,093,111	0	0	0	0
	Review workshop		n/a	n/a	n/a	n/a
	Material (a2)	2,730,000	n/a	n/a	n/a	n/a
	Mean of transportation price (b2)	50,000,000	n/a	n/a	n/a	n/a
	No of days (c2)	2	n/a	n/a	n/a	n/a
	Mean of transportation number (e2)	13	n/a	n/a	n/a	n/a

	Mean of transportation ULY (f2)	5	n/a	n/a	n/a	n/a
	Animal marking training		n/a	n/a	n/a	n/a
	Material (g2)	252,000	n/a	n/a	n/a	n/a
	Mean of transportation price (h2)	50,000,000	n/a	n/a	n/a	n/a
	No of days (i2)	14	n/a	n/a	n/a	n/a
	Mean of transportation number (j2)	1	n/a	n/a	n/a	n/a
	Mean of transportation ULY (k2)	5	n/a	n/a	n/a	n/a
For coordination	*Sub total7= o2/(360*p2)*l2*m2*n2*	*29,630*	n/a	n/a	n/a	n/a
	No of people (l2)	4	2	1		1
	No of working days (m2)	40	20	30		45
	Percentage time spent on vaccination (n2)	0.5	0.375	0.375		0.75
	Computer price (o2)	400,000	300,000	300,000		300,000
	Computer ULY (p2)	3	3	3	3	3

Table S13. Overhead calculation details

Vaccine distribution level		Central	Regional	Provincial	Communal	Private vets
Overheads = sub total1 + sub total2 + sub total3 + sub total4 + sub total5 + sub total6		58,328,343	167,964	121,917	202,709	1,183,517
For vaccine transportation	*Sub total1 = e + (f + g + h)*c*a + b*k + i*l*m + j*l*m + d*c*a*	*14,140,120*	*53,833*	*24,367*	*91,800*	*668,600*
	Supply number (a)	1	1	2	3	2
	Importing cost (b)	2.45	0	0	0	0
	Number of days of transportation (c)	12	1	1	0.5	1
	Mean of transportation daily maintenance cost (d)	4,167	833	833	200	150
	Fuel cost (e)	2,052,540	50,000	20,000	3,000	44,000
	Tool fees (f)	0	1,000	400	0	800
	Ice cost (g)	0	1,500	200	0	500
	Phone call cost per day for transportation (h)	1,000	500	750	500	1,250
	Fuel cost per day for field transportation (i)	n/a	n/a	0	1,750	2,000
	Field mean of transportation daily maintenance cost (j)	n/a	n/a	0	200	150
	Number of doses transported (k)	4,908,400	292,775	63,150	12,017	85,000
	No of days of transportation in the field (l)	n/a	n/a	n/a	45	48
	No of vaccination teams (m)	n/a	n/a	n/a	1	6
For vaccine storage	*Sub total2= n*o*p*q + u + (v/30)*t + s*t*r*	*18,899,285*	*8,381*	*5,238*	*32,809*	*47,527*
	Cold room number (n)	2	n/a	n/a	n/a	n/a
	Cold room maintenance daily cost (o)	1,667	n/a	n/a	n/a	n/a
	Cold room storage time in days (p)	360	n/a	n/a	n/a	n/a
	Percentage of use for PPR vaccine storage (q)	0.9	n/a	n/a	n/a	n/a
	Refrigerator number (r)	1	2	1	1	2
	Refrigerator maintenance daily cost (2.5%) (s)	104	*0*	*0*	*0*	35
	Refrigerator storage time in days (t)	90	40	50	65	70
	Power cost (u)	17,809,910	8,381	5,238	6,809	14,666
	Gas recharging cost per month (v)	0	*0*	*0*	12,000	12,000

For vaccine field delivery	*Sub total3 = z + y*x*w*	*0*	*0*	*0*	40,500	90,200
	No of vaccination teams (w)	n/a	n/a	n/a	1	6
	No of working days per team (x)	n/a	n/a	n/a	45	48
	Icebox price per day (y)	n/a	n/a	n/a	150	275
	Phone call (z)	n/a	n/a	n/a	33,750	11,000
For sensitization	*Sub total4 = b1*a1+c1*a1+d1*f1*a1+e1*	16,400,300	0	21,500	37,600	251,450
	No of session (a1)	1	n/a	2	8	13
	Phone call per session (b1)	100,000	n/a	0	500	1,500
	Fuel cost (c1)	2,822,000	n/a	10,000	2,000	5,000
	Mean of transportation maintenance cost (15%) (d1)	11,139	n/a	750	200	150
	Media broadcast costs (e1)	13,055,000	n/a	0	16,000	165,000
	Mean of transportation number (f1)	38	n/a	1	1	1
For supervision	*Sub total5 (central) = h1*g1*j1+i1+k1+l1*<br>*Sub total5 (others)* = h1*g1*j1+i1*j1+k1+l1*j1*g1	*2,822,028*	*79,750*	*44,813*	*0*	*36,500*
	No of days (g1)	5	1	1	n/a	1
	Phone call cost per day (h1)	2,057	1,000	1,200	n/a	1,000
	Fuel cost (i1)	2,052,450	13,000	6,750	n/a	2,500
	No of session (j1)	6	5	5	n/a	10
	Vaccine shipment (k1)	400,000	0	0	n/a	0
	Mean of transportation maintenance cost (15%) (l1)	307,868	1,950	1,013	n/a	150
For training and meetings	*Sub total6 = m1+n1+o1+p1+q1+r1+s1+t1+u1*	6,016,610	0	0	0	0
	Review workshop		n/a	n/a	n/a	n/a
	Phone call cost (m1)	200,000	n/a	n/a	n/a	n/a
	Fuel cost (n1)	1,986,000	n/a	n/a	n/a	n/a
	Room rent (o1)	0	n/a	n/a	n/a	n/a
	Media broad cast costs (p1)	1,000,000	n/a	n/a	n/a	n/a
	Mean of transportation maintenance cost (15%) (q1)	297,900	n/a	n/a	n/a	n/a
	Animal marking training		n/a	n/a	n/a	n/a
	Phone call (r1)	32,000	n/a	n/a	n/a	n/a

	Fuel cost (s1)	1,935,400	n/a	n/a	n/a	n/a
	Room rent (t1)	275,000	n/a	n/a	n/a	n/a
	Mean of transportation maintenance cost (15%) (u1)	290,310	n/a	n/a	n/a	n/a
For coordination	*Sub total7 = e2+j2+d2+f2*g2*a2+h2*i2*a2*	*50,000*	*26,000*	*26,000*	*0*	*89,240*
	Campaign duration (a2)	90	90	90	n/a	60
	No of people (b2)	4	2	1	n/a	1
	No of working days (c2)	40	20	30	n/a	45
	Transport cost (d2)	0	6,000	10,000	n/a	14985
	Communication cost (e2)	50,000		5,000	n/a	10,000
	Room rent (f2)	0	0	0	n/a	2,067
	PPR vaccination part (g2)	0	0	0	n/a	0.25
	Taxes dues (h2)	0	0	0	n/a	1,250
	PPR vaccination part (i2)	0	0	0	n/a	0.18
	Reporting phone call cost (j2)	0	20,000	11,000	n/a	19,750

Table S14. Vaccine wastage cost calculation details

Vaccine distribution level	Central	Regional	Provincial	Communal	Private vets
Total cost = a3-b3-c3	8,755,675	1,396,528	53,858	19,667	17,334
No of doses delivered (a3)	7,356,800	300,100	65,000	12,725	85,000
No of vaccinated animal (b3)	4,445,444	234,122	60,888	11,191	84,272
No of doses available (c3)	2,448,400	7,325	1,850	708	0
