# Supplementary material for: Peste des Petits Ruminants (PPR) Vaccination Cost Estimates in Burkina Faso
Source: Animals (Basel). 2022 Aug 22;12(16):2152. doi: 10.3390/ani12162152 (PMC9405497; doi:10.3390/ani12162152)
Supplement: Supplementary file 1 [file animals-12-02152-s001.zip › Tables S13-S21.pdf]

Cost calculation details by the nature of cost

Note: unit cost= total cost/number of animals vaccinated

- Variable cost
- Fixed cost

Table S13. Number of animals vaccinated in each level of vaccine distribution

Vaccine distribution level	Central	Regional	Provincial	Communal	Private vets
Number of vaccinated animals	4,445,444	234,122	60,888	11,191	84,272

Table S14. Vaccine purchase cost calculation details

Vaccine distribution level	Central	Regional	Provincial	Communal	Private vets
Fixed cost	0	0	0	0	0
Variable cost = a * b	105,846,022	0	0	0	0
Vaccine unit price (a)	23.81	n/a	n/a	n/a	n/a
Number of doses used (b)	4,445,444	234,122	60,888	11,191	84,272

Table S15. Details of fixed and variable vaccine transportation cost calculation

Vaccine distribution level		Central	Regional	Provincial	Communal	Private vets
Fixed cost		0	0	0	0	0
Variable cost = sub total1+ sub total2+ sub total3+ sub total4+ sub total5+ sub total6		15,237,453	125,438	85,547	140,199	966,989
	Number of supplies (a)	1	1	2	3	2
*Import cost (sub total1) = b*c*		*12,025,580*	*0*	*0*	*0*	*0*
	Unit import cost (b)	*2.45*	n/a	n/a	n/a	n/a
	Number of doses used (c)	4,908,400	292,775	63,150	12,017	85,000
*Mean of transportation cost (sub total2) = d/(e*360)*f*a + g*f*a*		*383,333*	*14,722*	*29,444*	*1,550*	*1,967*
	Mean of transportation price (d)	50,000,000	25,000,000	25,000,000	1,500,000	1,500,000
	Mean of transportation ULY (e)	5	5	5	5	5
	Number of days of transportation (f)	12	1	1	1	1
	Mean of transportation maintenance cost per day (g)	4,167	833	833	200	150
Fuel cost (sub total3)		*2,052,540*	*50,000*	*20,000*	*3,000*	*44,000*
*Personnel cost (sub total4) = i*k*h*a + i*j*h*a*		*764,000*	*57,667*	*33,333*	*9,500*	*56,667*
	No of people (h)	2	2	2	1	1
	No of days of transportation (i)	12	1	1	1	1
	Per diem (j)	23,500	20,500	0	0	15,000
	Daily salary (k)	8,333	8,333	8,333	6,333	13,333
*Field transportation cost (sub total5) = l/(m*360)*p*q + o*p*q + n*p*q*		*0*	*0*	*0*	*125,250*	*859,200*
	Motorbike cost (l)	n/a	n/a	n/a	1,500,000	1,500,000
	Motorbike ULY (m)	n/a	n/a	n/a	5	5
	Fuel cost per day (n)	n/a	n/a	n/a	1,750	2,000
	Field mean of transportation maintenance cost per day (o)	n/a	n/a	n/a	200	150
	No of days of field transportation (p)	n/a	n/a	n/a	45	48
	No of vaccination teams (q)	n/a	n/a	n/a	1	6
*Other cost (sub total6) = r/(s*360)*a*i+t*a*i+u*a*i+v*a*i*		*12,000*	*3,049*	*2,769*	*899*	*5,156*
	Cold box price (r)	0	35,000	25,000	71,420	20,000
	Cold box ULY (s)	2	2	2	2	2
	Tool fees (t)	0	1,000	400	0	800

	Ice (u)	0	1,500	200	0	500
	Phone call cost per day (v)	1,000	500	750	500	1,250

Table S16. Details of fixed and variable vaccine storage cost calculation

**Vaccine distribution level**		**Central**	**Regional**	**Provincial**	**Communal**	**Private vets**
Variable cost		0	0	0	0	0
Fixed = sub total1+ sub total2+ sub total3+ sub total4+ sub total5+ sub total6		23,474,285	30,603	19,127	51,918	87,551
*Sub total1 (Cold room cost) = b/(d*360)*a*e*f+c*a*e*f*		*5,580,000*	*0*	*0*	*0*	*0*
	No of cold room (a)	2	n/a	n/a	n/a	n/a
	Cold room building cost (b)	25,000,000	n/a	n/a	n/a	n/a
	Maintenance cost per day (c)	1,667	n/a	n/a	n/a	n/a
	Cold room ULY (d)	10	n/a	n/a	n/a	n/a
	Storage time in days (e)	360	n/a	n/a	n/a	n/a
	Percentage of use for PPR (f)	1	n/a	n/a	n/a	n/a
*Sub total2 (Refrigerator cost) = h/(j*360)*g*k + h*g*k*		*84,375*	*22,222*	*13,889*	*18,056*	*43,750*
	No of Refrigerators (g)	1	2	1	1	2
	Refrigerator price (h)	1,500,000	500,000	500,000	500,000	500,000
	Refrigerator maintenance cost per day (2.5%) (i)	104	0	0	0	35
	Refrigerator ULY (j)	5	5	5	5	5
	Storage time in days (k)	90	40	50	65	70
*Sub total3 (Electricity cost for central) = l*m*e*a*0.29+l*n*e*a*0.71*<br>*Sub total3 (Electricity cost for other levels) = l*m*k*g*		*17,809,910*	*8,381*	*5,238*	*6,809*	*14,666*
	No of Kwh/day (l)	224.2	0.97	0.97	0.97	0.97
	Cost of Kwh (peak moment) (m)[1]	165	108	108	108	108
	Cost of Kwh (full moment) (n)	88				
*Sub total4 (Gas cost) = =p/(360*q)*k + o*k*		*0*	*0*	*0*	*27,053*	*29,134*
	Gas recharging cost per month (o)	0			400	400

[1] *For the central level, the cost is Kwh depends on the electricity consumption moment in the days. It is estimated that 29% of electricity consumed in done during the peak moment (10:00AM -2:00PM and 4:00 PM – 7:00 PM) and 71 percent during the full moment (other hours).*

	Gas bottle price (p)	0	1	1	17,500	17,500
	Gas bottle ULY (q)	1	1	1	3	3

[1] For the central level, the cost is Kwh depends on the electricity consumption moment in the days. It is estimated that 29% of electricity consumed in done during the peak moment (10:00AM -2:00PM and 4:00 PM – 7:00 PM) and 71 percent during the full moment (other times).

Table S17. Details of fixed and variable Vaccine field delivery cost calculation

Vaccine distribution level		Central	Regional	Provincial	Communal	Private vets
Fixed cost = e*b*a		0	0	0	300,000	0
Variable cost = sub total1 + sub total2 + sub total3		0	0	0	967,275	4,541,361
*Sub total1 (Vaccinators cost for communal) =d*c*b*a + e*b*a*<br>*Sub total1 (Vaccinators cost for private vets) = (d*c*b*a) + e*		*0*	*0*	*0*	*870,000*	*4,213,600*
	No of vaccination teams (a)	n/a	n/a	n/a	1	6
	No of people per team (b)	n/a	n/a	n/a	2	2
	No of working per team (c)	n/a	n/a	n/a	45	48
	Daily salary (d)	n/a	n/a	n/a	6,333	-
	Per diem (e)	n/a	n/a	n/a	150,000	4,213,600
*Sub total2 (Vaccination material cost) = (f/g + h/I + j/k + l/m)*n*		*0*	*0*	*0*	*34,133*	*214,894*
	Vaccination syringe price (f)	n/a	n/a	n/a	30,000	30,000
	No of doses supported (g)	n/a	n/a	n/a	15,000	25,000
	Needle price (h)	n/a	n/a	n/a	250	250
	No of doses supported (i)	n/a	n/a	n/a	1,000	1,000
	Spare glass price (j)	n/a	n/a	n/a	3,000	3,000
	No of doses supported (k)	n/a	n/a	n/a	10,000	5,000
	Marking clamp (l)	n/a	n/a	n/a	10,000	10,000
	No of animal supported (m)	n/a	n/a	n/a	20,000	20,000
	No of animal vaccinated (n)	n/a	n/a	n/a	11,191	84,272
*Sub total3 (Other field cost) = o/(p*360)*a*c + q*a*c + r*			n/a	n/a	n/a	*95,533*
	Cold box price (o)	n/a	n/a	n/a	71,420	20,000
	Cold box ULY (p)	n/a	n/a	n/a	3	3

	Ice price per day (q)	n/a	n/a	n/a	150	275
	Phone call cost (r)	n/a	n/a	n/a	33,750	11,000
Sub total4 (Vaccine wastage)= s-t-u		*8,755,675*	*1,396,528*	*53,858*	*19,667*	*17,334*
	No of doses delivered (s)	7,356,800	300,100	65,000	12,725	85,000
	No of vaccinated animal (t)	4,445,444	234,122	60,888	11,191	84,272
	No of doses available (u)	2,448,400	7,325	1,850	708	0

Table S18. Details of fixed and variable sensitization cost calculation

**Vaccine distribution level**		**Central**	**Regional**	**Provincial**	**Communal**	**Private vets**
Variable cost		0	0	0	0	0
Fixed cost = sub total1 + sub total2 + sub total3		33,181,356	0	162,611	233,600	634,450
Personnel cost	*Sub total1 (central)= a*c*b*e + d*<br>*Sub total1 (provincial)= a*b*c*e + d*c*a*<br>*Sub total1 (communal and private vets) = a*b*c*e + d*a*	*10,775,500*	*0*	*113,333*	*161,333*	*274,667*
	No of people involved (a)	100	n/a	2	2	2
	No of days per session (b)	1	n/a	1	1	1
	No of sessions (c)	1	n/a	2	8	13
	Per diem (d)	9,775,500	n/a	20,000	30,000	55,000
	Daily salary (e)	10,000	n/a	8,333	6,333	6,333
Material and other costs	*Sub total2 (central) = i/(360*j)*c*b*k + l + h + g + f + m*<br>*Sub total2 (others) = I/(360*J)*c*b + l*c*b + h*c*b + g*c*b + f*c*b + m*	*9,350,856*	*0*	*49,278*	*56,267*	*194,783*
	Refreshment for farmers per session (f)	3,310,000	n/a	0	3,500	2,500
	Phone call per session (g)	100,000	n/a	0	500	1,500
	Fuel cost (h)	2,822,000	n/a	10,000	2,000	10,000
	Mean of transportation price (i)	50,000,000	n/a	25,000,000	1,500,000	1,500,000
	Mean of transportation price ULY (j)	5	n/a	5	5	5
	Mean of transportation No (k)	38	n/a	1	1	1
	Mean of transportation maintenance cost per day (15%) (l)	11,139	n/a	750	200	150
	Other materials (m)	1,640,000	n/a	0	0	0
	Sub total3 = n + o + p + q + r	13,055,000	0	0	16,000	165,000

Media broadcast costs	Town crier cost (n)	30,000	0	0	16,000	65,000
	Radio cost (o)	12,000,000	0	0	0	100,000
	Television cost (p)	600,000	0	0	0	0
	Newsletter cost (q)	250,000	0	0	0	0
	Other cost (interpreter and hostess) (r)	175,000	0	0	0	0

Table S19. Details of fixed and variable supervision cost calculation

**Vaccine distribution level**		**Central**	**Regional**	**Provincial**	**Communal**	**Private vets**
Variable cost		0	0	0	0	0
Fixed cost = sub total1 + sub total 2 + sub total 3		8,095,361	424,194	257,590	0	178,167
Personnel cost	*Sub total1 (central) = b*a*d*e + c*a*d*e*<br>*Sub total1 (others) = b*a*d*e + c*	*4,440,000*	*275,000*	*143,333*	*0*	*133,333*
	No of days (a)	5	1	1	n/a	1
	Daily salary (b)	10,000	8,333	8,333	n/a	13,333
	Per diem (c)	27,000	150,000	60,000	n/a	0
	No of people involved (d)	4	3	2	n/a	1
	No of sessions (e)	6	5	5	n/a	10
Transportation cost	*Sub total2 (central) = f/(360*g)*a*e + h + i*<br>*Sub total2 (others) = f/(360*g)*a*e+h/360*e*a+i*e*a*	*3,193,651*	*144,194*	*108,257*	*0*	*34,833*
	Mean of transportation cost (f)	50,000,000	25,000,000	25,000,000	n/a	1,500,000
	Mean of transportation cost ULY (g)	5	5	5	n/a	5
	Mean of transportation maintenance cost (15%) (h)	307,868	702,000	364,500	n/a	54,000
	Fuel cost (i)	2,052,450	13,000	6,750	n/a	2,500
Other costs	*Sub total 3 = j + k*	*461,710*	*5,000*	*6,000*	*0*	*10,000*
	Phone call cost (j)	61,710	5,000	6,000	n/a	10,000
	Vaccine shipment (k)	400,000	0	0	n/a	0

Table S20. Details of fixed and variable training and meetings cost calculation

**Vaccine distribution level**		**Central**	**Regional**	**Provincial**	**Communal**	**Private vets**
Variable cost		0	0	0	0	0
Fixed cost = sub total1 + sub total2		34,560,054	0	0	0	
Review workshops	*Sub total1 =b + d + e + f + g + h + I + c*a*k + j/(360*m)*l*k + n*	*12,007,122*	*0*	*0*	*0*	
	No of people (a)	80	n/a	n/a	n/a	n/a
	Per diem (b)	2,877,000	n/a	n/a	n/a	n/a
	Daily salary (c)	10,000	n/a	n/a	n/a	n/a
	Cocktail (d)	594,000	n/a	n/a	n/a	n/a
	Phone call (e)	200,000	n/a	n/a	n/a	n/a
	Fuel cost (f)	1,986,000	n/a	n/a	n/a	n/a
	Material (g)	2,730,000	n/a	n/a	n/a	n/a
	Room rent (h)	-	n/a	n/a	n/a	n/a
	Media broad cast cost (i)	1,000,000	n/a	n/a	n/a	n/a
	Mean of transportation price (j)	50,000,000	n/a	n/a	n/a	n/a
	No of days (k)	2	n/a	n/a	n/a	n/a
	Mean of transportation No (l)	13	n/a	n/a	n/a	n/a
	Mean of transportation ULY (m)	5	n/a	n/a	n/a	n/a
	Mean of transportation maintenance (15%) (n)	297,900	n/a	n/a	n/a	n/a
Animal marking training	*Sub total2 = b + d + e + f + g + h + I + c*a + j/(360*m)*l*k + n*	*22,552,932*	*0*	*0*	*0*	
	No of people (a)	163	n/a	n/a	n/a	n/a
	Per diem (b)	16,369,000	n/a	n/a	n/a	n/a
	Daily salary (c)	6,333	n/a	n/a	n/a	n/a
	Cocktail (d)	1,978,000	n/a	n/a	n/a	n/a
	Phone call (e)	32,000	n/a	n/a	n/a	n/a
	Fuel cost (f)	1,935,400	n/a	n/a	n/a	n/a
	material (g)	252,000	n/a	n/a	n/a	n/a
	Room rent (h)	275,000	n/a	n/a	n/a	n/a
	Media broad cast cost (i)	-	n/a	n/a	n/a	n/a
	Mean of transportation price (j)	50,000,000	n/a	n/a	n/a	n/a
	No of days (k)	14	n/a	n/a	n/a	n/a

	Mean of transportation No (l)	1	n/a	n/a	n/a	n/a
	Mean of transportation ULY (m)	5	n/a	n/a	n/a	n/a
	Mean of transportation maintenance (15%) (n)	290,310	n/a	n/a	n/a	n/a

Table S21. Details of fixed and variable coordination cost calculation

**Vaccine distribution level**		**Central**	**Regional**	**Provincial**	**Communal**	**Private vets**
Variable cost		0	0	0	0	0
Total cost = sub total1 + sub total2 + sub total3		4,509,630	155,167	122,875	0	548,625
Personnel cost	*Sub total1 = d*b*a*c*	*800,000*	*125,000*	*93,750*	*0*	*450,000*
	No of people in the coordination (a)	4	2	1	n/a	1
	No of working days (b)	40	20	30	n/a	45
	Percentage time spent on PPR vaccination (c)	0.50	0.38	0.38	n/a	0.75
	Daily salary (d)	10,000	8,333	8,333	n/a	13,333
Reporting cost	*Sub total2 =g*e*h + f*e*h + i*	*3,680,000*	*20,000*	*11,000*	*0*	*19,750*
	No of people in reporting (e)	11	n/a	n/a	n/a	n/a
	Per dem (f)	5,000	n/a	n/a	n/a	n/a
	Daily salary (g)	10,000	n/a	n/a	n/a	n/a
	No of working days (h)	22	n/a	n/a	n/a	n/a
	Phone call cost (i)	50,000	n/a	n/a	n/a	n/a
Other costs	*Sub total3 =p*q*j + r*s*j + m/(360*n)*a*b*o + k + l*	*29,630*	*10,167*	*18,125*	*0*	*78,875*
	Campaign duration (j)	90	90	90	n/a	60
	Transportation cost (k)	0	6,000	10,000	n/a	333
	Phone call cost (l)	0	0	5,000	n/a	10,000
	Computer cost (m)	400,000	300,000	300,000	n/a	300,000
	Computer ULY (n)	3	3	3	n/a	3
	Computer percentage use for PPR (o)	0.50	0.38	0.38	n/a	0.75
	Room rent (p)	n/a	n/a	n/a	n/a	2,067
	PPR vaccination % in the room rent (q)	n/a	n/a	n/a	n/a	0.25
	Taxes dues (r)	n/a	n/a	n/a	n/a	1,250
	PPR vaccination % in the turnover (s)	n/a	n/a	n/a	n/a	0.18
