# Supplementary material for: Peste des Petits Ruminants (PPR) Vaccination Cost Estimates in Burkina Faso
Source: Animals (Basel). 2022 Aug 22;12(16):2152. doi: 10.3390/ani12162152 (PMC9405497; doi:10.3390/ani12162152)
Supplement: Supplementary file 1 [file animals-12-02152-s001.zip › Tables S22 and S23.pdf]

Total values calculation method

Table S22. Total cost calculation in the public channel

Component cost	Central	Regional	Provincial	Commune	Total cost	
Component1	a	b	c	d	T1= a*0.6 + b + c +d	(63)
Component n	f	g	h	i	Tn= f*0.6 + g + h +i	(64)
Total					T1+T2…+Tn	(65)

- 0.6 0.6 (60%) is the percentage of the animal vaccinated in the public channel
- a, b,…i are the average cost at a given distribution level

Table S23. Total cost calculation in the private channel

Component cost	Central	Private vets	Total cost	
Component1	a	b	T1= a*0.4 + b	(66)
Component n	c	d	Tn= c*0.4 + d	(67)
Total			T1+T2…+Tn	(68)

- 0.4 (40%) is the percentage of the animal vaccinated in the private channel
- a, b,… are the average cost at a given distribution level
